# Design Thinking Innovation Within the Quadruple Helix Approach: a Proposed Framework to Enhance Student Engagement Through Active Learning in Digital Marketing Pedagogy

**DOI:** 10.1007/s13132-022-00984-1

**Published:** 2022-03-02

**Authors:** John R. T. Bustard, Daniel Hsiang Hsu, Rachael Fergie

**Affiliations:** Department of Management, Leadership and Marketing, Ulster University, Belfast, Northern Ireland

**Keywords:** Design thinking, Co-creation; Quadruple Helix, Active learning, Student engagement, Curriculum design

## Abstract

The purpose of this paper is to develop a framework for enhancing student engagement through active learning and design thinking workshops online. The COVID-19 pandemic has increased the need for digital engagement exponentially, yet the important experiences of collaborative and active learning (AL) have become more challenging to facilitate and support in circumstances of remote access during classes. As such, design thinking (DT) online presented a unique opportunity to explore this collaborative approach to engaging in user-centred design and design innovation. To explore a specific curriculum design dilemma and validate the methodology adopted, online DT workshops were conducted with two different cohorts of students toward co-creating more student-centred forms of learning in two digital marketing (DM) courses. The approach was guided by the POLARIS active learning framework to embed the framework’s seven perspectives (Purpose, Objectives, Learning landscape, Activities and assessment, Resources, Inter-relation with stakeholders, and Student engagement) which were considered to better enhance student engagement in an online learning environment. Using the framework proposed, which additionally integrates Quadruple Helix Model (QHM) actors, would facilitate further DT innovation towards co-creating new value in digital marketing pedagogy, curriculum design, and beyond.

## Introduction

Digital marketing (DM) pedagogy in university teaching is focused on creating future-ready, digitally competent marketers. DM educators may share the same mission as ourselves, to best prepare graduates who possess the strategic ability to apply tactics effectively to the subject matter discipline, and this teaching–learning objective is an essential component in modern marketing pedagogical design (Shah et al., 2019). Most importantly, it is clear that approaches to ‘learning by doing’ or active learning (AL) as it is more commonly understood have become a cornerstone of developing future-ready marketers at both undergraduate and postgraduate levels of study to maintain subject relevance and interest in a more competitive and evolving learning environment (Loh & Ang 2020). Critically, this approach is seen that ‘when students believe what they are doing is important, to their studies and future profession, they are more engaged in class’ (Kahu & Nelson, 2018, p. 63). It is therefore that the application of AL and its effect on student engagement and learning development could offer a significant opportunity to educators in higher education.

The COVID-19 pandemic has created unique circumstances whereby engagement using digital mechanisms has become integral to success but without the input of students, many projects may not deliver. That said, engaging students in AL undoubtedly presented a significant challenge in the context of a pandemic due to the requirement for learners to be taught fully online (Mishra et al., 2020). In the light of these challenges, focus was required toward more innovative delivery to support students who were experiencing less peer support given the lack of person-to-person interactions. For many in higher education, while it has taken time in adapting to online and flipped modes of teaching and learning toward engaging students through online platforms (Rathner & Schier, 2020), finding standardised procedures to this evolved method of practice has resulted in a need for further development. With this in mind, the authors applied design thinking (DT) in an innovative manner to better involve students online, as stakeholders, partners, and co-creators, at this evolving interface of value co-creation in higher education (Dollinger & Lodge, 2020). Indeed, further stakeholder engagement through additional integration of the Quadruple/Quintuple Helix model framing offers to support future pedagogic innovation and thinking in higher education (see Morawska-Jancelewicz, 2021), particularly the pursuit of Society 5.0 and Industry 5.0 challenges such as sustainability (Carayannis & Morawska-Jancelewicz, 2022).

In developing pedagogical competencies, the methodology of DT has been successfully applied in the context of teaching marketing research (see Zarzosa, 2018) and DM (see Schiele & Chen, 2018). Nevertheless, in this instance, and fully online, the DT approach for this paper focused on how students might utilise a wide range of MarTech or marketing technology to support active and deeper learning in higher education within a pandemic environment. The overall aim of this paper is to develop a framework for enhancing student engagement through online collaboration leveraging different digital platforms toward value co-creation. The paper is divided into four sections; first, we discuss DM pedagogy and how the challenges could be turning into opportunities; second, we present the methodology used in this paper including the innovation process of DT through the development of the POLARIS active learning framework (Purpose, Objectives, Learning landscape, Activities and assessment, Resources, Inter-relation with stakeholders, and Student engagement) to better enhance student engagement in an online learning environment; third, we outline the steps for reframing pedagogy through DT workshops; finally, we discuss the implications and draw on conclusions of the adaptability of this DT innovation towards co-creating new values in DM pedagogy, curriculum design, and beyond.

## Digital Marketing Pedagogy: from Challenges to Opportunities

DM educators may share the same mission as ourselves to help students demonstrate skills in the effective use of digital technology to solve business challenges and exploit opportunities of the digital age. In our DM curriculum design and delivery, we aim to cover a significant range of elements and thus to understand the subject’s breadth of learning in digital strategy, search engine optimisation (SEO), content marketing, social media marketing, email marketing, and marketing automation (Chaffey & Ellis-Chadwick, 2019). While much of this can be explored in lectures, given the context of the COVID-19 pandemic, the biggest teaching–learning challenge was the lack of ability to collaborate with students effectively in virtual settings. In addition, students may experience less peer support due to the lack of in-person interaction in online classes throughout their studies. A significant challenge of many of the courses being re-engineered due to the pandemic presents further challenges in terms of support for each individual student during the remote learning process. For example, the cohort sizes for our DM courses completing at both the undergraduate and postgraduate levels were relatively large, with over 90 students and over 60 students each year respectively. Fortunately, the movement toward online delivery was not fully untested in higher education and platforms and processes were often in place to support student online engagement (Mishra et al., 2020). However, technology adoption and finding suitable approaches to this evolved method of practice have resulted in a need for further experience development.

To find better ways to involve students as stakeholders, partners, and co-creators, the authors employed the form of active and deeper learning for students in a DM course. According to Rathner and Schier (2020), the power of AL is that students engage more in building their knowledge and understanding of their own learning and are more likely to achieve the specified learning outcomes in response to AL classroom activities and resources provided by educators. In a similar vein, Wanner (2015) aligns to this perspective by stating AL as ‘the extent to which students are involved in experiences that involve actively constructing new knowledge and understanding’ (p. 155). Although integrating students in AL presented a further challenge within the context of a pandemic, an online platform was developed aiming for better student engagement at www.MarTech-Laboratory.com. This student-led website was envisioned as a community hub to connect current and future students as well as alumni to support active and deeper learning through the wider implications of marketing technology (known as MarTech) tools and resources. Moreover, the DT approach was adapted to engage students beyond the classroom continuously leveraging online tools and techniques to inform the development of supplemental digital learning resources and supporting educators in knowing the progress of students through a unique dashboard linked to certifications of learning delivered by a 3rd party in the MarTech domain.

This continuous project-based approach has also been supported by a different student cohort—these more as stakeholders, partners, and co-creators helping to develop new ideas in partnership with teaching staff and to collectively integrate this with course purpose and outcomes. This activity is envisaged to be applied each academic year to promote AL toward better student engagement within our DM pedagogical design, wrapping around the course experience. This approach is enabled through DT workshops conducted as part of an ongoing development of a student-led online platform which supports a real-world experience of marketing technologies meeting the demands of all learners. More specifically, the DT workshops were carried out through the online platform MURAL (a large visual collaboration platform for problem solving and communication) and supported through Blackboard Collaborate. This evolved method of online collaboration enabled full implementation of DT processes on a remote basis. In addition, the online approach facilitated the stages of DT effectively, and the interactions of participants in these stages, who were able to co-create ideas mirroring the active manner of offline DT workshops. As an engagement process, DT requires time to establish an appropriate stakeholder group to coordinate a viable time for workshop delivery. In this instance, students were recruited from prior DM classes and from inter-disciplinary areas, including those involved in a student-led consulting society at the University, which could contribute different insights into the curriculum redesign process. These different insights are important to innovative thought and as a means to engage wider stakeholders in DT. As such the AL model prescribed for the workshops integrates the Quadruple Helix Model (QHM) toward developing richer AL experiences informed by a wider stakeholder group.

The QHM has been used to provide for the integration of important actors within wider ecosystems engaged in pursuits toward new innovations (Leydesdorff, 2012). The QHM of innovation has been regarded as significant in the conception of embedding universities as key actors within networks of forward-looking and feedback-driven stakeholders, driven by broader framing of innovation challenges (Carayannis & Campbell, 2011). More recently, the QHM has been adapted to increasingly diverse contexts where further consideration of human centredness in designing smart systems is requiring new and refreshed thinking (Carayannis et al., 2021). The QHM can therefore be adopted as a framing or superset of government, industry, civil society, university, and more laterally through the Quintuple Helix Model the environment. Critically, all conceptions of the QHM are aimed toward regulating the interaction amongst stakeholders with the aim to increase knowledge creation and sharing (Morawska-Jancelewicz, 2021). The QHM thus provides an important opportunity for developing further stakeholder integration into the context of higher education learning and as such is considered a key element of the following proposed AL model.

To appropriately frame this pedagogically and to provide some parameters of the future teaching and learning experience, the POLARIS active learning framework was adopted to guide ‘prototyping’ within the time available and to assist learners toward understanding AL and engaging creatively with AL as a learning solution (Bustard et al., 2021). More specifically, the POLARIS acronym acts to integrate key understanding around seven core perspectives of course delivery: Purpose, Objectives, Learning landscape, Activities and assessment, Resources, Inter-relation with stakeholders, and Student engagement. Informing this design, Mizokami (2018, p. 89) proposes ‘six practical suggestions to enhance the quality of AL-based instruction: (1) assessing learning hours outside the class, (2) backward design, (3) curriculum development, (4) multiple classes per week, (5) building an environment for active learning, and (6) the flipped classroom’. Building on this by leveraging DT as a process towards employing pedagogical innovation, the authors aimed to better integrate AL for enhancing student engagement as a result. Although the notion of student engagement has been considered from several perspectives in the past, for the purpose of this paper, our focus is placed through the lens of student engagement considered by Kahu (2013; Kahu & Nelson, 2018) pivotal insights on this important and dynamic teaching and learning nexus in higher education. Kahu’s work considers the core experience at the educational interface, aligned with its influences and outcomes, with a critical focus on students’ affective, cognitive, and behavioural experience.

## Design Thinking Methodology

DT can be defined as ‘a human-centred systems thinking approach that creates experiences for stakeholders by matching human factors with technological feasibility and business viability’ (Meinel & Plattner, 2009, p. 904). As a process of innovation, organisations and individuals applying DT are ‘directed toward new integrations of signs, things, actions, and environments that address the concrete needs and values of human beings in diverse circumstances’ (Buchanan, 1992, p. 21). This development of DT has been of growing interest in its application extending to many fields beyond solely product innovation. Focusing on user insights, the utilisation of DT in this paper ultimately engaged stakeholders toward developing effective solutions—on how to enhance student experience/engagement in learning the DM courses supported by the MarTech-Laboratory platform. Moreover, the approach is achieved through encouraging emphasis on human experience toward addressing the needs of those who will engage with the service (Carroll, 2015). In this way, it is ensured that the authors adopted a design ‘by’ approach, where the potential user groups of the online platform were actively involved in the design process of their own learning platform (Kaulio, 1998).

According to Henriksen et al. (2017), DT can be applied creatively to educational problems and help address challenges that educators are faced with. In this sense, the workshop stemming from this paper aids educators to reach beyond the traditional way of curriculum design/planning and develop a more agile approach in planning engagement opportunities. It does so by using evaluation methods at an earlier stage so that educators can achieve higher levels of impact by engaging key stakeholders early during the curriculum design process (Wolfe, 2019). Although DT is not considered to be a linear approach, it is commonly classified through five distinct stages, namely empathising, defining, ideating, prototyping, and testing (Brown, 2009), all of which were implemented within our DT workshops. That said, participants in the workshop were focused toward developing new value creation opportunities by leveraging the MarTech-Laboratory platform as a support to the DM courses at both undergraduate and postgraduate levels of study. Following completion of the workshops, the educators reviewed outputs provided by the student cohorts and actioned those items that were prioritised by the group and viable for inclusion in the development of the platform.

### The Innovation Process of Design Thinking

Using the Quadruple Helix Model (QHM), our DT approach was further developed by embedding its innovative perspectives and applicability as a conceptual lens for inter-relating key stakeholders (Carayannis & Campbell, 2009). The QHM has been applied to successfully engage stakeholders in research processes at the nexus of policy, industry, society, and academia toward developing relationships through open innovation (Miller et al., 2018). Adapting these key stimuli and perspectives, it offers opportunities for students to consider key stakeholder in value co-creation of knowledge and to effectively integrate them into the process of curriculum design (Stier & Smit, 2021). This was achieved by engaging with students in considering how perspectives of policy, industry, society, and academia can integrate as experience components at the educational interface. As such, the experience of students and/or stakeholders at this interface can further be considered an enabler in terms of elements of the experience such as how value can be increased through the affective, cognitive, and behavioural realms of engagement (Kahu, 2013). The POLARIS active learning framework was therefore proposed to ensure that the benefits of a multi-stakeholder approach to open innovation in classroom experiences could be applied to enhance better student engagement in supporting societal challenges (with consideration of the UN’s sustainable development goals). More specifically, the acronym acts to frame the process of aligning the DT approach in the delivery of the QHM-inspired experiences:

- Purpose,
- Objectives,
- Learning landscape,
- Activities and assessment,
- Resources,
- Inter-relation with stakeholders through the QHM, and
- Student engagement.

The DT workshops were initiated in April 2021 and delivered live online over a 3-h period simultaneously via Blackboard Collaborate and using the MURAL platform for further collaboration, with focus on the DM courses at both undergraduate and postgraduate levels of study. Through the process, the educators were able to engage with the students immerse their learning experiences at the University, in varying degrees with the given questions:

- Beyond supporting the course outcomes, what purpose can the platform serve?
- Beyond the learning handbook, what outcomes could be delivered supported by the platform?
- What are the key building blocks or processes of learning DM that can be supported via the platform?
- What are the best ways to engage on key aspects of the learning landscape through AL supported by the platform? What are the assessment opportunities beyond, and what is currently in place that could leverage the platform?
- What resources or automations could further support learning via the platform?
- What ways can we engage the QHM stakeholders to add value to the experience via the platform?
- How can we improve student engagement through the platform?

**Fig. 1:**
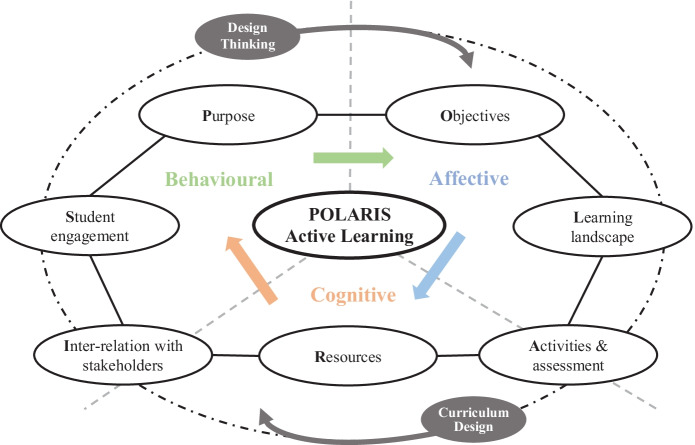
The POLARIS active learning framework within the Quadruple Helix approach

Figure 1 presents the POLARIS active learning framework within the Quadruple Helix approach. The innovation process of DT was then guided by the framework to better enhance student engagement in an online learning environment.

## Reframing Pedagogy Through Design Thinking Workshops

**Fig. 2:**
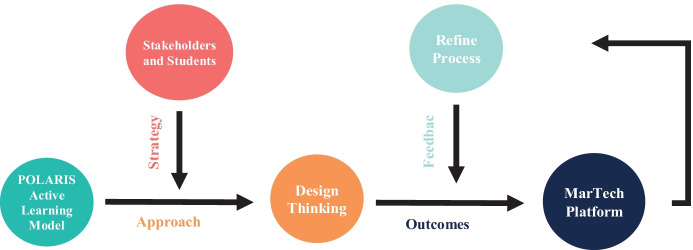
The process of reframing the DM pedagogy through the DT workshops

To explore a specific curriculum design dilemma and validate the methodology adopted, the online DT workshops were conducted with two different cohorts of students for co-creating more active learning and the QHM motivated experiences in two DM courses. There were 14 students engaged to reflect on the current learning experience and through the DT approach, aided by a pedagogical framing of AL using the POLARIS framework, they were tasked with designing in the QHM informed pedagogic innovations. Focus was placed on enhancing student engagement toward a curriculum redesign process that best supports higher value engagement with a focus on generating positive affective, cognitive, and behavioural outcomes across its delivery (Hartikainen et al., 2019). Figure 2 presents the process of reframing the DM pedagogy through the DT workshops. As discussed earlier in the paper, two online workshops were held and ran simultaneously, one focusing on an undergraduate DM course and the other on a postgraduate DM course. The following discusses the six steps on how the 3-h DT workshops were delivered.

### Step 0: Pre-workshop (Experience Design Building on Feedback)

Prior to the workshop, a student feedback survey was conducted, in order to create insights to support successful experience design. This served to gather feedback from the students who had previously experienced the DM course and was toward assessing their general learning experience against Kahu’s (2013) conceptual framework of student engagement, related through affective, cognitive, and behavioural impacts. This initial step was useful in guiding the development and iteration of the MarTech-Laboratory platform toward supporting the user-learners’ experience. Table 1 presents student feedback on the course experience, and the AL components, which were also supported through the MarTech-Laboratory platform. This assisted in clarifying some of the enablers and barriers to student engagement across sub-themes of cognitive, affective, and behavioural aspects.

**Table 1:** Pre-workshop: student feedback on the course experience (aligned to Kahu, 2013; Kahu & Nelson, 2018)

Engagement		Outcomes	
		Positive	Negative
**Affective**	Enthusiasm	‘This was the most practical module throughout entire university experience therefore I was eager to start this module. John was enthusiastic throughout the 12 weeks and was a great help which made this module easier. I gained some great insight into digital marketing into this module and believe this is one of the most important modules in the entire degree’	‘The assignment should not have been a group one given the current COVID situation… The Strategic assignment was turned into an individual one due to the circumstances, this should have been the same’
	Interest	‘I enjoyed the project as it was real world and similar to what we would undertake’	‘Live lectures over pre-recorded would help keep students interested and engaged’
**Cognitive**	Self-Regulation	‘I enjoyed how we were able to find useful websites, tools and applications to use within our coursework. I also enjoyed how we were able to gain additional accreditations through inbound marketing on HubSpot’	‘In the beginning we were taught things for a later assessment instead of our first assessment. Better lay out of information would have been appropriate’
	Deep Learning	‘Great application of digital marketing platforms etc.—for any students that didn’t get hands-on experience with various platforms and tools via placement, this would be exceptionally useful. The module consecrated my module and gave context to the work I did on placement’	‘A similar module should perhaps have been offered in year 2 to prepare students for the real-life applications of marketing tech’
**Behavioural**	Participation	‘Engaging with real clients is very important for my learning. As someone with an interest in consulting as well the assignment was a great way to apply skills. I have found myself applying the learning from this module in the real world’	‘More information on the structure of the assignment, the only guidance provided was a past students example. I would also advise against group work in the middle of a global pandemic, especially not one that is worth 75% of a final year module’
	Time & Effort	‘The assessment order and due dates helped stay focused as they were convenient…’	‘Didn’t understand why we had to complete an external course (HubSpot), minimal guidance for the assignment was given’
	Interaction	‘…content was excellent and very informative. Each class was interesting and the use of Nearpod made the learning experience enjoyable’	‘Group work was difficult’

The following section presents the DT steps which were then applied within the workshops and provides some context in terms of process, time, and sought outcomes. Step 5 highlights the novel integration of the POLARIS framework with the embedded element of inter-relating through the QHM. POLAIRS offers a useful macro-, meso-, and micro-perspective of requirements from purpose and outcomes at macro-level to inter-relation of the QHM stakeholders and student engagement opportunities as the micro-level.

### Step 1: Overview of Experience of Engaging with DM Courses

Empathising is core and foundational to DT as a human-centred approach. The authors outline two stages of this practice of its application. First, focus was placed on the participants toward building a better understanding of their learning experience of the course. A 1-h briefing session followed by Q&A was held prior to the DT workshop. The aim was to introduce the MarTech-Laboratory platform and immerse more clearly in the needs of student learning within the context of a pandemic. Second, for the workshop session, the participants were asked to identify challenges through a short 10-min exercise situating focus on their own learning experience from empathic perspectives. This understanding of user-learner experience of the courses would later be revisited and refocused on whether the online platform, through application of MarTech, could better support student learning experience and student engagement.

### Step 2: Create Proto-Persona’s Profile to Align to Motivations and Needs of the Students

The defining stage focused on the use of the insights formed through the first step of the workshop toward defining the learner journey throughout the DM course experience. During this step, the participants were asked to co-develop proto-persona’s (user profiles). This helped encapsulate the challenges faced across the experience timeline by these example users and allowed for reflection against critical performance aspects and ascertaining key moments that are critical to successful outcomes in the learner’s journey. The participants were also encouraged to add images for the imagined personas to support visualising the user’s profile. Through the DT problem identification stage, they were able to add their ideas by using virtual colourful sticky notes via the MURAL platform which allows for online collaboration and visual problem solving. This method supported the participants to discuss the focus on the learners’ behaviours and actions, needs, and pain points as well as to better understand demographic and psychographic anchors impacting learners on the course during this initial stage. This assured focus from which to design with empathy.

### Step 3: Complete the Journey Map Aligned to the Identified Stages of the Course

To further define the user/learners’ experience, the participants were asked to complete a journey map by adding additional sticky notes of their ideas-solutions to the MURAL board online. This step had required them to identify the different stages of the experience journey based on the personas they had identified. For example, the ‘stage one’ aspect was what students perceived as the first thing that happened in their course experience journey in DM. Through this exercise, the participants were able to effectively discuss the actual and possible feelings (both positive and negative) and pain points as well as opportunities at each identified point of the experience journey. The participants were then able to assess the contributions through group dialogue to explore innovative opportunities for the MarTech-Laboratory platform to further support or enhance based on deeper understanding explored through discussion (Carroll, 2015).

### Step 4: Brainstorm the Ideation Session on a ‘How Might We’ Question

In the ideating stage, exploration of a divergent range of ideas/solutions was considered at an individual level firstly (in quiet and with little distraction) and then collectively. The participants were firstly asked to brainstorm the question on ‘how might we’ use the platform and MarTech tools (e.g. bots, automation, and personalisation) to enhance student engagement and experience with the course. Individually, the participants first silently brainstormed ideas and placed them onto the MURAL board with their own-selected coloured sticky notes. Next, they worked collaboratively to group the ideas by thematic topics or similarities. Finally, in the group brainstorming area, the notes were prioritised by individuals using three own-selected coloured dots per participant which they dragged and dropped to their preferred ideas, establishing the most popular concepts. At this stage of the workshop, agreement was sought toward prioritising ideas, features, and inputs for the MarTech-Laboratory platform. These final considerations were managed by participants against constraints of budget, time, and personnel based on prioritising best return for learners.

### Step 5: Alignment to the POLARIS Active Learning Framework

The outputs from previous steps were then used to guide the participants toward a rapid prototyping process. This was achieved by considering and debating opportunities aligned to the POLARIS framing in order to link to important aspects of embedding AL at a macro-, meso-, and micro-level for their learning experience journey. The participants were asked to have a group discussion on each aspect by using the sticky notes on the MURAL. Building on this and by leveraging DT as a process toward pedagogical innovation, the authors aimed to better integrate AL and enhance student engagement as a result through leveraging the MarTech-Laboratory platform. Student engagement has been considered from several perspectives in the past, but in this paper, focus has been placed through Kahu (2013; Kahu & Nelson, 2018) conceptual framework, which considers the core experience at the educational interface. This interface is framed by its influences and outcomes, with a critical focus on students’ affective, cognitive, and behavioural experience at its core. Indeed, Fig. 1 best represents this interplay across DT, guided by POLARIS toward developing positive student engagement and inter-relating QHM opportunities to integrate policy, industry, societal or academic actors, elements, or insights to enrich DM pedagogy.

### Step 6: Post-workshop (Experience Design Building on Student Feedback)

The final step saw testing of the MarTech-Laboratory platform by the participants after a period of 4 weeks and following the inclusion of updates to the platform based on those suggested ideas and features agreed in the prior stage. The participants reflected on the overall solution and provided feedback on the prototype platform and shared ideas for the designers to consider iterating the platform further prior to launch. More specifically, the participants were sent out a survey asking for their views on the updated platform and related to the adapted site post-workshop prototyping. When asked ‘Will the user understand the point of this site? (Consider as a student undergrad, postgrad or enterprise)’, the answers were overwhelmingly positive which is an important aspect to clarify. In relation to their answers to the question ‘Is the site easy to navigate?’, the participants again agreed that ‘yes’ it was. Most importantly, when asked ‘How would you change the site to meet the learner’s needs even more?’, Table 2 presents the responses to this query from seven student respondents.

**Table 2:** Student feedback on potential development post-workshop iterations

Student (alias name)	Comments on how you would change the site to meet the learner’s needs even more
Nina	Not at all I think the amount of detail it provides for students is extremely comprehensive. It is a great resource for any student of marketing no matter what level
Andy	Maybe a welcome video on the home page from a current or past student explaining the website’s purpose
Barry	There is lots of support on the website, and helpful links, this will help the student experience
Tom	I would perhaps just optimise the promotion of the courses section more even with a news reel somewhere on the homepage to get direct traffic from there to the courses page as it is something that I would personally be extremely engaged with. This could be a very unique perspective as it may not be why the majority of students will use this site
Joe	Offer more options for the students for consolidation
Alex	I was going to say somewhere to leave questions but as the bot is there, I guess it is not necessary! I think it is obvious all the feedback from the session was taken on board and the site looks great!
David	I would continue to build out different forms of informative content, I already see the Blog posts, embedded podcasts and certificates so continuing to provide plenty of resources like this will help students succeed

It is noted that the authors aim to repeat all the steps supported by a different student and stakeholder cohort each academic year toward successful co-creation for continuously enhancing student engagement and learning development in an online AL environment. The aim is to create a virtuous cycle of iteration and integration of important insights from the QHM actors or elements integrated through AL processes toward enhanced student engagement.

## Implications and Conclusions

This paper proposes the POLARIS framework and its inter-relation of QHM as a guide toward DT innovation, where students and stakeholders can co-create new values in digital marketing pedagogy, curriculum design, and beyond toward supporting better outcomes for Industry and Society 5.0 (Carayannis & Morawska-Jancelewicz, 2022).

Developing a more robust approach to ensuring courses remain strategic, contemporary, creative, and innovative is key to developing future-ready graduates. The process of DT offers potential opportunities to higher education practitioners to develop engaging student learning experiences. However, it is worth noting that DT is vulnerable to certain levels of unpredictability and to challenges based on the less defined nature of the outcomes. Thus, own biases from the facilitators and participants must be considered and accounted for at an early stage of the process. In this instance, some pre-testing with trusted stakeholders would support assuring such an approach to develop awareness of the limitations of the process from the outset. This is so that both facilitators and participants can understand what is possible and achievable within the parameters of DT during the workshop (such as in this example, where constraints relating to already existent course specifications exist). In addition to this, there is a necessity for facilitators to allow DT as a process to unfold in a structured but unconstrained manner, to develop participation from all stakeholders and ensure empathy lead on the desire of quality of student engagement (fundamental to DT’s success).

Beyond these aspects, it is critical that the process of a curriculum redesign is supported appropriately with the people, time, data, and resources required to complete. In this instance, DT was used for a complicated redesign task to integrate a more integrated learning experience supported through a web-based platform. If DT is to be applied for other pedagogical needs, it is important to be clear on the intent of the process and to communicate with stakeholders appropriately.

In terms of the potential adaptation of DT in higher education contexts, this research experience offered valuable insights into enhancing student engagement embedded within AL in module design. That said, focus on the online platform in our example may have impeded potential creativity benefits to wider curriculum design opportunities. It is therefore realistic to consider that DT could be applied toward other user contexts to offer even more significant benefits as a form of value co-creation. Using the framework proposed, it would provide an important guide to support macro-, meso-, and micro-understanding of DT’s focus and purpose toward enhancing student engagement at the educational interface through such co-creation. Here are two areas where the authors feel that this evolved DT approach could be adapted to help student engagement in achieve learning outcomes in embedding AL:

### Curriculum Redesign to Embed AL

Focusing in on curriculum design, particularly as part of a revalidation effort, the proposed framework would offer an important starting point to engage a course team as they initiate their course development activities. A 3-h workshop could be focused on the development of the purpose, objectives, learning landscape, activities and assessment, resources, inter-relation with QHM stakeholders, and how to enhance student engagement in online or face-to-face or hybrid learning. Educators can then leverage this approach in a pre- or post-course delivery setting to share the students’ learning experience of a cohort. This would provide support toward gaining insights from students and stakeholders and the state of the art and how it could be further developed through wider stakeholder insights and through design, curriculum development as focus and AL as conduit to enhance student engagement and course delivery.

### DT Through POLARIS as Stakeholder Engagement Activity

Course leaders and/or key faculty members could also leverage DT through POLARIS to better integrate thinking of stakeholders from across broader areas of influence from outside (economic, social, political, etc.) and within academia (e.g. digital learning) who can add values into the curriculum and pedagogy. Furthermore, the evolved DT approach framed against the backdrop of a course, experience element, and educative process would offer a refreshing and invigorating teaching–learning experience at the quadruple helix of industry, policy, academia, and society. Indeed, adopting the sustainability agenda and its principles within the QHM might be an essential means to maintain subject relevance in higher education, particularly with the impetus placed on the UN’s Sustainable Development Goals in recent years. Thus, student engagement in this evolved DT approach which leverages the POLARIS active learning framework provides an adaptable means through which to purposefully co-create new values aligned to a curriculum, with students and stakeholders of digital marketing pedagogy, curriculum design, and beyond.

## Data Availability

The data that support the findings of this paper are openly available from the corresponding author, JRTB, upon reasonable request. The platform developed in its current form can be visited at www.MarTech-Laboratory.com.
